# Liposome-Coupled Peptides Induce Long-Lived Memory CD8^+^ T Cells Without CD4^+^ T Cells

**DOI:** 10.1371/journal.pone.0015091

**Published:** 2010-11-30

**Authors:** Maiko Taneichi, Yuriko Tanaka, Terutaka Kakiuchi, Tetsuya Uchida

**Affiliations:** 1 Department of Safety Research on Blood and Biological Products, National Institute of Infectious Diseases, Tokyo, Japan; 2 Department of Immunology, Toho University School of Medicine, Tokyo, Japan; New York University, UNITED STATES

## Abstract

CD8^+^ T cells provide broad immunity to viruses, because they are able to recognize all types of viral proteins. Therefore, the development of vaccines capable of inducing long-lived memory CD8^+^ T cells is desired to prevent diseases, especially those for which no vaccines currently exist. However, in designing CD8^+^ T cell vaccines, the role of CD4^+^ T cells in the induction and maintenance of memory CD8^+^ T cells remains uncertain. In the present study, the necessity or not of CD4^+^ T cells in the induction and maintenance of memory CD8^+^ T cells was investigated in mice immunized with liposome-coupled CTL epitope peptides. When OVA-derived CTL epitope peptides were chemically coupled to the surfaces of liposomes and inoculated into mice, both primary and secondary CTL responses were successfully induced. The results were further confirmed in CD4^+^ T cell-eliminated mice, suggesting that CD4^+^ T cells were not required for the generation of memory CD8^+^ T cells in the case of immunization with liposome-coupled peptides. Thus, surface-linked liposomal antigens, capable of inducing long-lived memory CD8^+^ T cells without the contribution of CD4^+^ T cells, might be applicable for the development of vaccines to prevent viral infection, especially for those viruses that evade humoral immunity by varying their surface proteins, such as influenza viruses, HIV, HCV, SARS coronaviruses, and Ebola viruses.

## Introduction

It has been reported by numerous investigators that CD4^+^ T cells are essential for the maintenance of memory CD8^+^ T cells [1]–[5]. However, in the induction and maintenance of CD8^+^ memory T cells, different roles of CD4^+^ T cells have been described [6]–[9]. In the so-called “classical model”, CD4^+^ T cells contribute to memory CD8^+^ T-cell generation indirectly via APCs [6]. Through the CD40-CD40L interaction between CD40L on CD4^+^ T cells and CD40 on APCs, CD4^+^ T cells “license” APCs for the induction of memory CD8^+^ T cells. As an alternative to this APC licensing model, Bourgeois et al. [7] provided evidence demonstrating that CD4^+^ T cells contribute directly to CD8^+^ T cells through CD40 on CD8^+^ T cells, rather than indirectly via APCs. However, these findings were countered by studies in which long-lived CD8^+^ memory T cells were generated in the absence of CD40 expression on CD8^+^ T cells [8], [9]. In addition, as for the role of CD40-CD40L interaction in the induction of memory CD8^+^ T cells, Hernandez et al. [10] reported that CD8^+^ T cells themselves provided CD40L in order to license APCs for the induction of memory CD8^+^ T cells. In their scenario, although the CD40-CD40L interaction between T cells and DCs is indispensable for the induction of memory CD8^+^ T cells, CD4^+^ T cells are not necessarily involved. Thus, the research so far has not resolved the role of CD4^+^ T cells in the induction and maintenance of memory CD8^+^ T cells, although resolving this issue is a critical step in designing better vaccination and immunotherapeutic strategies.

Upon natural infection, the host responds by inducing humoral and cellular immunity against the pathogen. Humoral immune responses are represented by the production of antibodies that bind to the surfaces of bacteria and viruses, whereas cellular immune responses mediate immunity to intracellular pathogens. In general, extracellular antigens are presented via MHC class II molecules to CD4^+^ T cells, whereas intracellular antigens are presented via MHC class I molecules to CD8^+^ T cells. To induce antigen-specific CTL, antigens must be loaded onto the class I MHC processing pathway in APCs via cross-presentation [11]. In the cross-presentation, exogenous proteins cross over to the endogenous pathway to gain access to MHC class I molecules. Using this phenomenon, a generation of antigen-specific CTL responses might be useful in the development of vaccines that can prevent viral diseases. However, the currently approved alum adjuvant, which was first described by Glenny et al. [12] in 1926 and until today remains the only adjuvant approved for clinical use, is known to be effective only for the induction of humoral immunity, not for the induction of cell-mediated immunity [13]–[16]. Consequently, the development of a novel vaccine adjuvant is essential for the induction of cell-mediated immunity.

We previously reported that surface-coupled liposomal antigens could be presented by APCs to CD8^+^ T cells via MHC class I molecules if certain lipid components were chosen for the liposomes [17]. This antigen preparation was expected to be applicable for the development of tumor vaccines to induce antitumor responses and for the development of viral vaccines to induce virus-specific CTLs that effectively eliminate virus-infected cells [18]. Since the liposomal conjugates induced CTLs efficiently when CTL epitope peptides were coupled to the surfaces of liposomes [17], the liposomal conjugatesare expected to be applicable for the development of CTL-based peptide vaccines. In the development of peptide vaccines, it is essential to know whether a T helper epitope peptide is necessary for the induction of long-lived memory CD8^+^ T cells, an important step in vaccine preparation. This study was aimed at evaluating the role of CD4^+^ T cells in the induction of long-lived memory CD8^+^ T cells by liposome-coupled peptides.

## Results

### Induction of antigen-specific primary CD8^+^ T cells and CTLs in mice by OVA_257-264_-liposome conjugates

**Figure 1:**
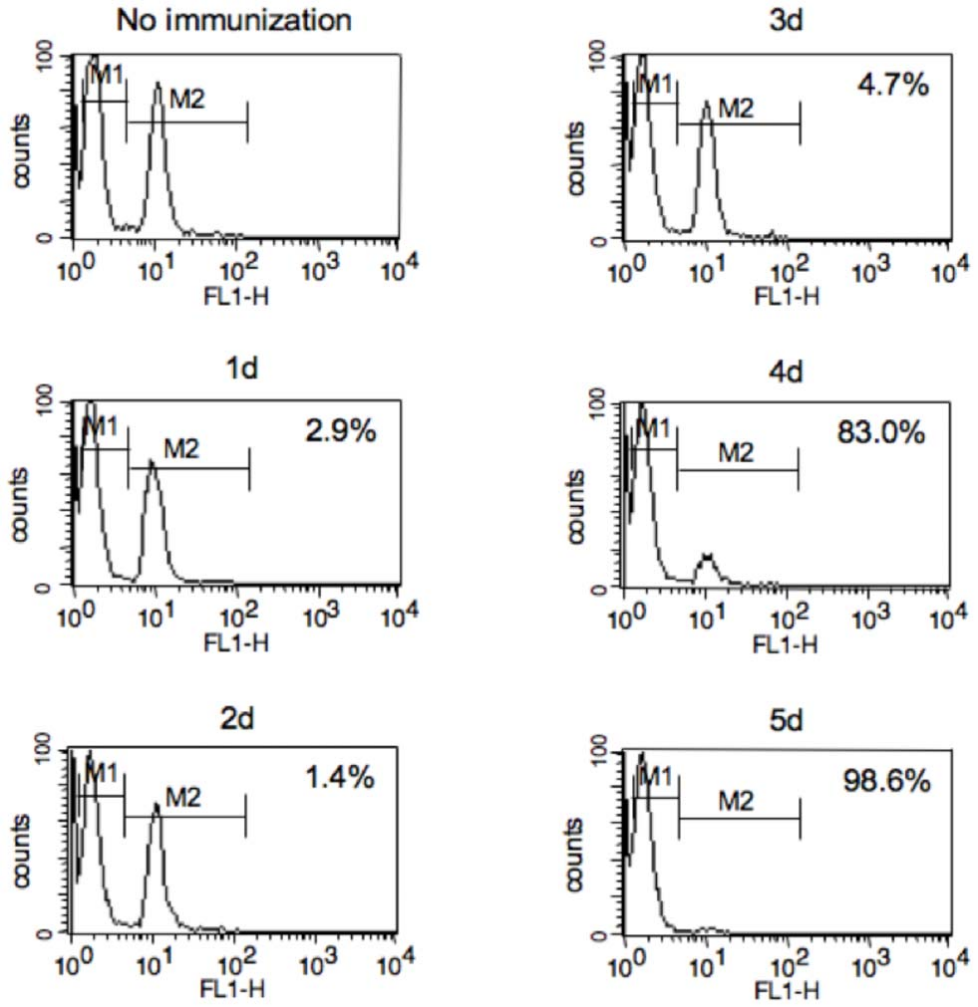
Kinetics of primary CTL response induced by OVA_257-264_-liposome conjugates. Mice were immunized with 50 µl of OVA_257-264_-liposome in the presence of 5 µg CpG; one to 5 days later, an *in vivo* CTL assay was performed as described in Materials and Methods. The numbers for each time period indicate percentages of target cells killed. Data are representative of three individual mice in each group for which similar results were obtained.

**Figure 2:**
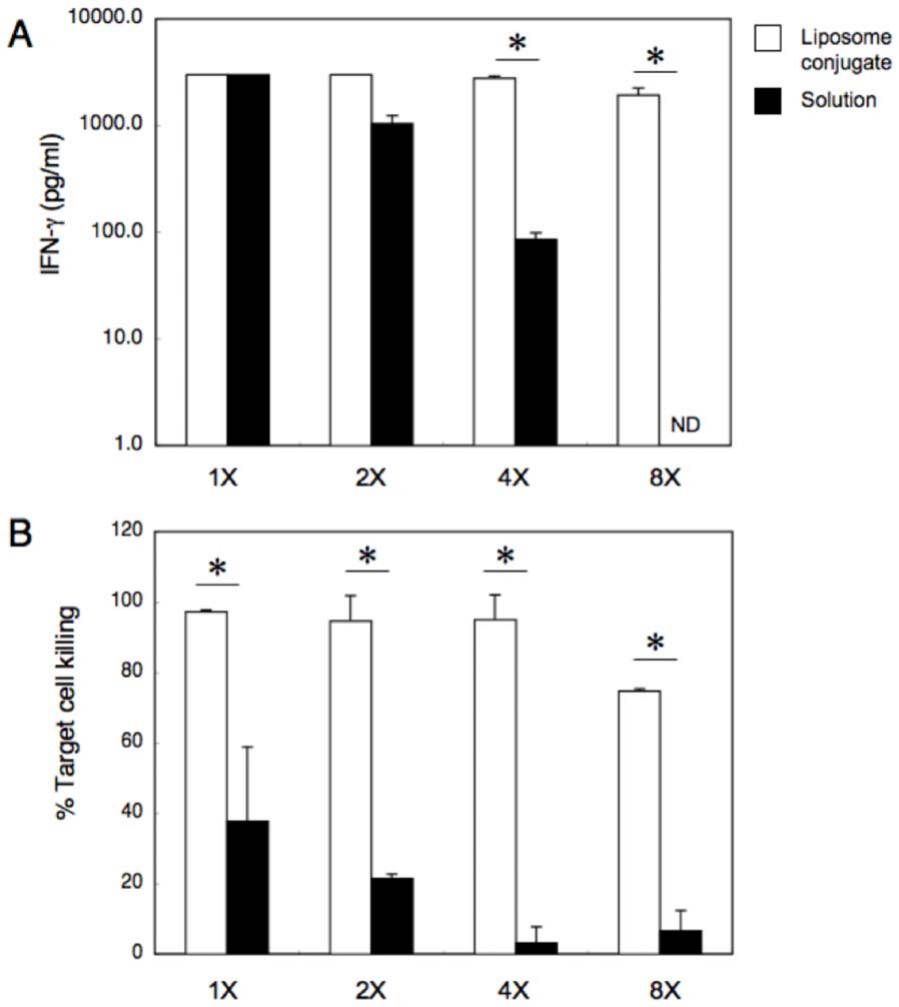
Dose-response of cytokine production by CD8^+^ T cell and CTL induction in mice immunized with OVA_257-264_-liposome or with OVA_257-264_ solution. A serial two-fold dilution of OVA_257-264_-liposome (open box) and OVA_257-264_ solution (closed box) were made in PBS, and mice were immunized with the diluents in the presence of 5 µg CpG. OVA_257-264_ solution containing equal amounts of peptides as those in OVA_257-264_-liposome. One week after the immunization, IFN-γ production by CD8^+^ T cells (**A**) and the CTL response (**B**) were monitored as described in Materials and Methods. Data represent means and SE of three mice per group. *, significant difference (p>0.01).

Mice were immunized with OVA_257-264_-liposome conjugates in the presence of CpG as described in Materials and Methods. A significant induction of CTL specific for OVA_257-264_ was observed on day 4 and a complete cell killing was observed as early as 5 days after the immunization (Figure 1). Therefore, in the following experiments, primary CTL responses were monitored at 7 days after immunization. Mice were then immunized with serially diluted solution of OVA_257-264_-liposome conjugates containing 0.3 (8×) to 2.4 µg (1×) of peptides or OVA_257-264_ solution that contained equal amounts of peptides as those in liposomal conjugates. Although OVA_257-264_-liposome and OVA_257-264_ solution seemed to induce a comparable level of T-cell cytokine production at the highest dose (2.4 µg/injection), a dose-dependent decrease was observed in mice immunized with OVA_257-264_ solution but not in mice immunized with OVA_257-264_-liposome, suggesting that OVA_257-264_-liposome was more effective than OVA_257-264_ solution in the induction of antigen-specific CD8^+^ T cell cytokine production (Figure 2A). Similar results were observed in T cell cytokine production; a dose of OVA_257-264_-liposome as low as 0.6 µg/mouse (4× dilution) induced a perfect killing as assayed by *in vivo* CTL assay, while OVA_257-264_ solution induced only a partial killing even at the highest dose (Figure 2B).

### Secondary CTL response in mice immunized with OVA_257-264_-liposome

**Figure 3 pone-0015091-g003:**
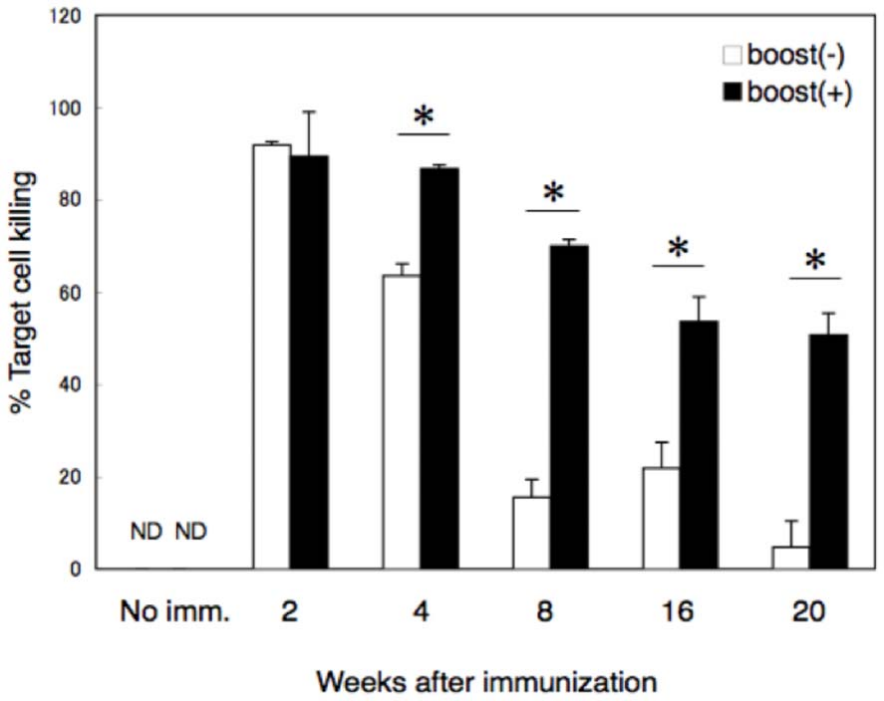
Secondary CTL response in mice immunized with OVA_257-264_-liposome. Mice were immunized with 50 µl of OVA_257-264_-liposome in the presence of 5 µg CpG, and 2, 4, 8, 16, and 20 weeks later, they received a booster ip injection with 200 µl of 1 mg/ml OVA in PBS (closed box) or no booster injection (open box). Three days after the booster injection, *in vivo* CTL assay was performed. Data represent mean percentages of cells killed and SEs of three mice per group. ND, not detected. *, significant difference (p>0.01).

**Figure 4 pone-0015091-g004:**
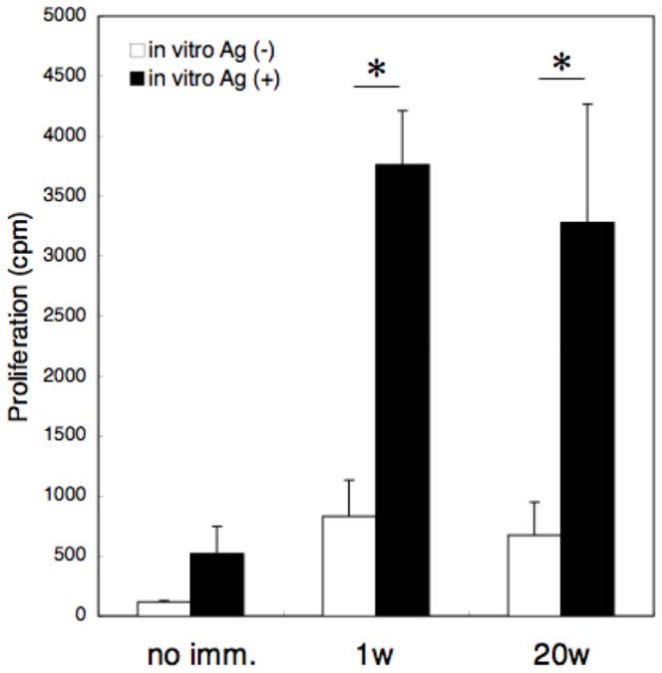
Antigen-specific CD8^+^ T-cell proliferation assay. Mice were immunized with OVA_257-264_-liposome and 1 week or 20 weeks later, CD8^+^ T cells of the immunized mice were cultured in the presence (closed box) or absence (open box) of OVA as described in Materials and Methods. Data represents mean ^3^H- thymidine incorporation and SE of triplicate cultures. *, significant difference (p>0.01).

Induction of secondary CTL responses in mice immunized with OVA_257-264_-liposome was further investigated. Mice were immunized with 50 µl of OVA_257-264_-liposome and 2, 4, 8, 16, and 20 weeks later, the mice received a booster injection with OVA. Three days after the booster injection, OVA_257-264_-specific cell killing was monitored. As shown in Figure 3, a complete cell killing was observed at 2 weeks after the immunization without a booster injection and, up to 20 weeks after the immunization, a significant recall response was observed upon booster injection with OVA. Inoculation of naive mice with the same dose of OVA as the booster injection (“No imm.” in Figure 3) did not induce a detectable CTL response. Interestingly, a significant recall response was observed even at 20 weeks when the primary CTL response was nearly undetectable. An antigen-specific CD8^+^ T-cell proliferation assay further confirmed the results; as shown in Figure 4, CD8^+^ T cells of mice immunized with OVA_257-264_-liposome significantly proliferated upon *in vitro* stimulation with OVA even 20 weeks after immunization.

### Effect of in vivo elimination with CD4^+^ T cells on the induction of long-lived memory CD8^+^ T cells by OVA_257-264_-liposome conjugates

**Figure 5:**
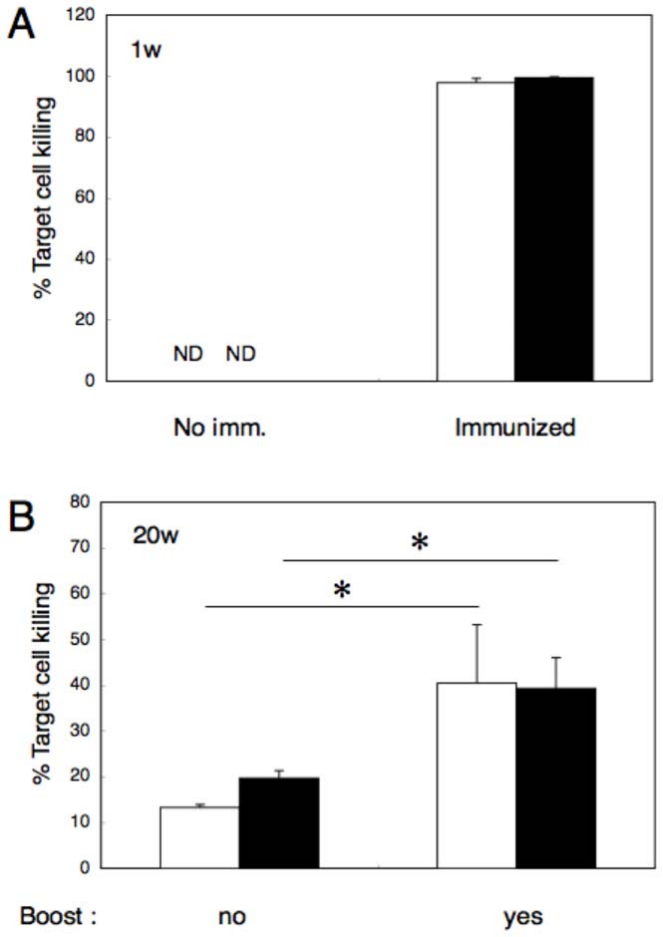
Effect of *in vivo* elimination of CD4^+^ T cells on the induction of primary and secondary CTL responses by OVA_257-264_-liposomes. Mice with (closed box) or without (open box) CD4^+^ T-cell elimination were immunized with 50 µl of OVA_257-264_-liposome solution in the presence of 5 µg CpG, and CTL induction was monitored. **A**, CTL response 1 week after immunization. **B**, CTL response 20 weeks after immunization with or without booster injection. *In vivo* CTL assay was performed 3 days after the booster injection. Data represent mean percent killing and SE of three mice per group. ND, not detected. *, significant difference (p>0.01).

To eliminate CD4^+^ T cells, mice were inoculated with GK1.5 as described in Materials and Methods, and immunized with OVA_257-264_-liposome. As shown in Figure 5, *in vivo* elimination with CD4^+^ T cells affected neither for primary (Figure 5A) nor for secondary (Figure 5B) CTL responses; even at 20 weeks after the immunization, a significant recall response, comparable to that in normal mice, was observed in mice from which CD4^+^ T cells had been eliminated.

## Discussion

In the present study, the role of CD4^+^ T cells in the induction and maintenance of memory CD8^+^ T cells was evaluated in mice immunized with liposome-coupled CTL epitope peptides. Although the inclusion of CpG, a ligand of TLR-9, was needed for the induction of the primary CTL response by OVA_257-264_-liposome, CD4^+^ T cells were not required in either primary or secondary response, since long-lived memory CD8^+^ T cells were readily induced only by immunization with CTL epitope peptides coupled to liposomes (Figures 3 and 4). This finding was further confirmed in CD4^+^ T cell-depleted mice (Figure 5). These results are in agreement with those reported previously by numerous investigators that CD4^+^ T cells are dispensable for the primary expansion of CD8^+^ T cells and their differentiation into cytotoxic effectors [2], [3], [5]. However, most of these researchers have claimed that secondary CTL expansion is wholly dependent on the presence of T helper cells during, but not after, priming [1]–[5].

We previously reported that surface-linked liposomal antigens induced IgE-selective unresponsiveness [19]. The results were consistent even when different coupling procedures for the antigens with the liposomes were employed [20]. During the course of an investigation intended to clarify the mechanism of IgE-selective unresponsiveness induced by surface-coupled liposomal antigens, we discovered an alternative approach to regulating the production of IgE, one that is independent of the activity of T cells [21]. Immunization of mice with OVA-liposome conjugates induced IgE-selective unresponsiveness without apparent Th1 polarization. Neither interleukin-12 (IL-12), IL-10, nor CD8^+^ T cells participated in the regulation. Further, CD4^+^ T cells of mice immunized with OVA-liposome were capable of inducing antigen-specific IgE synthesis in athymic nude mice immunized with alum-adsorbed OVA. On the other hand, immunization of the recipient mice with OVA-liposome did not induce anti-OVA IgE production, even when CD4^+^ T cells of mice immunized with alum-adsorbed OVA were transferred. In the secondary immune response, OVA-liposomes enhanced anti-OVA IgG antibody production but did not enhance ongoing IgE production, suggesting that the IgE-selective unresponsiveness induced by the liposomal antigen involved direct effects on IgE but not IgG switching *in vivo*. These results suggest the role of an alternative mechanism, one not involving T cells, in the regulation of IgE synthesis, and raise the possibility that the surface-linked liposomal antigens are potentially applicable for the development of novel vaccines with minimal induction of IgE synthesis. Moreover, given the relatively low allergic response to and increased antigenicity of the allergen, this form of antigen preparation would be applicable for allergen immunotherapy [22].

The potential usefulness of surface-linked liposomal antigens for application to vaccine development was further investigated. During the course of this investigation, a significant difference was observed in the recognition of liposomal antigens by antigen-presenting cells (APCs) between liposomes with different lipid components [23], and this difference was closely correlated with the adjuvant activity of liposomes [24]. In addition to this “quantitative” difference between liposomes with different lipid components, a “qualitative” difference (i.e., different abilities to induce cross-presentation) was also observed between liposomes with different lipid components [17]. Although the precise mechanism underlying this difference is currently unclear, the significant difference in membrane mobility observed between these liposomes [24] might affect their ability to induce cross-presentation. Thus, by utilizing their ability to induce cross-presentation, surface-linked liposomal antigens could be used to develop virus vaccines that induce a cytotoxic T-cell (CTL) response, as well as tumor vaccine preparations that present tumor antigens to APCs and induce effective antitumor responses [18].

Regarding the necessity of CD4^+^ T cells in the generation of memory CD8^+^ T cells, the results of the present study differed from those reported previously [1]-[5]. The difference in these findings may be due to differences in how mice were primed with antigens; in most of the studies reported previously, mice were primed by infecting viruses, such as LCMV [1], [3], [5], H3N2 influenza virus [2], and recombinant vaccinia virus [4], whereas in the present study, mice were immunized with OVA-derived CTL epitope peptides. Perhaps the difference in the requirements of CD4^+^ T cells observed among those studies [1]–[5] and the present study was due to the difference in the efficiency of inducing the presentation of the immunodominant CTL epitope by APCs. In general, only ∼1/2000 of the peptides in a foreign antigen expressed by an appropriate APC achieve immunodominant status with a given class I allele [25]. However, in the present study, immunization with OVA_257-264_-liposome successfully induced both primary and secondary CTL responses without the presence of CD4^+^ T cells (Figures 2 to [Fig pone-0015091-g003] [Fig pone-0015091-g004] 5). In addition, it was reported previously that antigens coupled to the surface of liposomes are recognized effectively by APCs and presented to T cells [24]. Therefore, although the TLR-ligand (CpG, in the present study) was necessary to mimic viral infection in order to induce CTL responses in the immunization with liposome-coupled peptides, CD4^+^ T cells were not required for the induction and maintenance of CD8^+^ memory T cells.

There is considerable interest in developing vaccines that elicit effective antiviral CD8^+^ T cell responses [26] against a variety of viruses, such as HIV [27], HCV [28], and SARS coronavirus [29]. For this purpose, the utilization of the immunodominant CTL epitope would be more effective than the use of an attenuated, inactivated, or subunit vaccine in the development of virus vaccines to elicit effective antiviral CD8^+^ T cell responses. For example, although the risk of a major global pandemic of avian influenza has created widespread concern, vaccines designed to induce antibodies against H5 haemagglutinin are expected to possess little or no efficacy, given the high rate of diversification of H5N1 strains due to the antigenic drift caused by point mutation of genes [30]–[33]. On the other hand, it is known that cytotoxic T cells specific for the internal proteins NP and M1 show high cross-reactivity between strains and between subtypes, reflecting high conservation of the internal proteins [34]–[37]. In addition, Lee et al. [38] recently reported that people who have not been exposed to H5N1 viruses have cross-reactive CD8^+^ T cell memory to a wide range of H5N1 peptides. Therefore, these peptides are expected to be used to add a CD8^+^ T cell component to current antibody-focused vaccine strategies with a view to reducing the impact of infection with novel influenza A viruses [39]. Epstein et al. [40] studied DNA vaccination in mice with plasmids expressing conserved nucleoprotein (NP) and matrix (M) from an H1N1 virus. However, the DNA vaccination alone protected poorly against a highly virulent strain of H5N1 influenza viruses.

Recently, we reported that peptides derived from the internal NP protein of the H3N2 influenza virus, chemically coupled to the surface of liposomes, induced antigen-specific CTLs and successfully inhibited the growth of H3N2 influenza virus in the lung [41]. More recently, we determined human HLA class I-restricted, immunodominant CTL epitopes derived from internal proteins of H5N1 influenza viruses [42]. Similar to those results reported previously [34]–[37], [43], most of the CTL epitopes determined were well conserved and were identical with those involved in H1N1 and H3N2 influenza viruses. The combined use of these CTL epitope peptides, common to influenza viruses, and the surface-linked liposomal antigens which induce long-lived memory CD8^+^ T cells without CD4^+^ T cell help, was demonstrated to be applicable for the development of a CTL-based influenza vaccine that is capable of inducing protection against heterosubtypic influenza viruses [42].

Taken together, these results suggest that surface-linked liposomal antigens might be applicable for the development of CTL-based vaccines to induce long-term prevention against infection with viruses other than influenza viruses, especially for those viruses that evade humoral immunity by varying their surface proteins, such as HIV, HCV, and SARS coronaviruses.

## Materials and Methods

### Mice

CBF1 mice (5–6 wk of age) were purchased from SLC (Shizuoka, Japan). All mice were maintained under specific pathogen-free conditions. Experiments in the present study were approved (permit numbers 208021 and 209082) by the Animal Research Committee of National Institute of Infectious Diseases, Tokyo, Japan and the mice were handled according to international guidelines for experiments with animals.

### Chemicals

All phospholipids were obtained from NOF Co. (Tokyo, Japan). Reagent grades of cholesterol were purchased from Wako Pure Chemicals (Osaka, Japan).

### Antigens and Reagents

Ovalbumin (OVA, grade VII) was purchased from Sigma-Aldrich. Mouse MHC class-I (K^b^)-binding peptides OVA_257-264_ (SIINFEKL) were obtained from Operon Biotechnologies (Tokyo, Japan). Synthetic CpG ODN (5002: TCCATGACGTTCTTGATGTT), phosphorothioate-protected to avoid nuclease-dependent degradation, was purchased from Invitrogen.

### Liposomes

The liposomes used in this study are provided by NOF corporation (Tokyo, Japan). They consisted of dioleoyl phosphatidylcholine (DOPC), dioleoyl phosphatidyl ethanolamine (DOPE), dioleoyl phosphatidyl glycerol (DOPG), and cholesterol in a 4∶3∶2∶7 molar ratio. The crude liposome solution was passed through a membrane filter (Nucleopore polycarbonate filter; Coster) with a pore size of 0.2 µm.

### Coupling of OVA peptides to liposomes

Liposomal conjugates with OVA peptides were prepared essentially in the same way as described previously [17] via disuccinimidyl suberate (DSS). Briefly, a mixture of 10 ml of anhydrous chloroform solution containing 0.136 mM DOPE and 24 µl of TEA was added in drops to 26.6 ml of anhydrous chloroform solution containing 0.681 mM DSS and stirred for 5 h at 40°C. The solvent was evaporated under reduced pressure, and 18 ml of a 2∶1 mixture of ethyl acetate and tetrahydrofuran was added to dissolve the residue. Then, 36 ml of 100-mM sodium phosphate (pH 5.5) and 90 ml of saturated NaCl aqueous solution were added to the solution, shaken for 1 min, and allowed to separate. To remove undesirable materials, the upper layer was washed with the same buffer and, after evaporation of the solvent, 3 ml of acetone was added to dissolve the residue. One hundred ml of ice-cold acetone was added in drops and kept on ice for 30 min to precipitate. Crystals were collected and dissolved in 5 ml of chloroform. After evaporation, 34.4 mg of DOPE-DSS was obtained. Then, 0.18 mM DOPC, 0.03 mM DOPE-DSS, 0.21 mM cholesterol, and 0.06 mM DOPG were dissolved in 10 ml of chloroform/methanol. The solvent was removed under reduced pressure and 5.8 ml of phosphate buffer (pH 7.2) was added to make a 4.8% lipid suspension. The vesicle dispersion was extruded through a 0.2-µm polycarbonate filter to adjust the liposome size. A 2-ml suspension of DSS-introduced liposome and 0.5 ml of 5-mg/ml OVA peptide solution were mixed and stirred for 3 days at 4°C. The liposome-coupled- and uncoupled peptides were separated as described above using CL-4B column chromatography. The resulting solution of OVA_257-264_-liposome conjugates contained 47 µg/ml of peptides as assessed by amino-acid quantitative analysis done by Toray Research Center (Kanagawa, Japan).

### Immunization

All the mice were immunized with indicated doses of OVA_257-264_-liposome conjugates via subctaneous injection in the presence of 5 µg/mouse CpG. For the booster immunization, the mice were immunized intraperitoneally (ip) with 200 µl of 1-mg/ml OVA in PBS solution.

### *In vivo* elimination of CD4^+^ T cells

For the *in vivo* elimination of CD4^+^ T cells, mice received weekly ip injection with 0.5 mg of GK1.5, a monoclonal anti-CD4 antibody, throughout the experimental period. This treatment resulted in a >99% decrease in the number of CD4^+^ T cells in the spleen and lymph nodes as determined by fluorescence-activated cell sorter (FACS) analysis.

### *In vivo* cytotoxicity assay

Spleen cells of naive CBF1 mice were labeled with either 0.5 µM (dull) or 5 µM (bright) CFSE for 15 min at 37°C using a Cell Trace CFSE cell proliferation kit (Molecular Probes, Eugene, OR) and washed twice with ice-cold PBS. CFSE-bright cells were subsequently pulsed with 0.5 µg/ml of OVA_257-264_ for 90 min at 37°C. CFSE-bright cells and CFSE-dull cells were mixed at a 1∶1 ratio, and then a total of 1×10^6^ cells was injected i.v. into the indicated group of mice. Twenty hours later, spleen cells were harvested from each mouse and analyzed by using FACSCalibur (Becton Dickinson, Mountain View, CA).

### Cell culture

All incubations were performed in RPMI-1640 (Invitrogen Life Technologies) supplemented with 10% heat-inactivated FCS (HyClone), 100 U/ml penicillin, and 100 µg/ml streptomycin (Invitrogen).

### Preparation of dendritic cells (DC) and CD8^+^ T cells

DCs and CD8^+^ T cells were obtained from spleen cells of CBF1 mice using the magnetic cell sorter system MACS according to the manufacturer's protocol using anti-CD11c and anti-CD8 antibody-coated microbeads (Miltenyi Biotec), respectively. CD8^+^ T cells and DCs were suspended in RPMI-1640 containing 10% FCS at cell densities of 2×10^6^/ml and 8×10^5^/ml, respectively. The CD8^+^ T cell suspension was plated at 250 µl per well onto 48-well culture plates (No. 3047; BD Biosciences), and 250 µl of DC suspension and 500 µl of 40 µM OVA_257-264_ solution in the same medium were added to the plates. After incubation in a CO_2_ incubator for 5 days, the culture supernatants were collected and assayed for the concentration of IFN-γ.

### Cytokine assays

IFN-γ in the culture supernatant was measured using the Biotrak mouse ELISA system (GE Healthcare, UK). All test samples were assayed in duplicate, and the SE in each test was always less than 5% of the mean value.

### T cell proliferation assay

Splenic CD8^+^ T cells (5×10^5^ cells/well) of immunized mice and whole spleen cells (1×10^5^ cells/well) of 25 Gy-irradiated naïve mice were cultured in 96-well plates for 4 days in the presence (closed box) or absence (open box) of 20 µM OVA. The cells were pulsed with 1.25 µCi (0.046 MBq) [^3^H]-thymidine (PerkinElmer) for the final 6 hours of the culture, and, after harvesting, cell proliferation was monitored using TopCount (PerkinElmer).

### Statistical analysis

Student's *t* test was employed for the statistical analysis.
